# Role of mTOR and VEGFR Inhibition in Prevention of Metastatic Tumor Growth in the Spine

**DOI:** 10.3389/fonc.2020.00174

**Published:** 2020-02-19

**Authors:** Tobias Kratzsch, Andras Piffko, Thomas Broggini, Marcus Czabanka, Peter Vajkoczy

**Affiliations:** ^1^Department of Neurosurgery, Charité University Hospital, Berlin, Germany; ^2^Department of Physics, University of California, San Diego, La Jolla, CA, United States

**Keywords:** spinal metastases, targeted therapy, preclinical mouse model, everolimus, axitinib

## Abstract

**Objective:** Spinal metastatic disease remains a major problem of oncological diseases. Patients affected may suffer pain, spinal instability, and severe neurological deficits. Today, palliative surgery and radiotherapy are the mainstays of therapy. In contrast, preventive treatment strategies or treatment concepts for an early stage are lacking. Here, we have used a syngeneic, experimental spine metastases model in the mouse to test the efficacy of mTOR inhibition and anti-angiogenesis on the formation and progression of spinal melanoma metastases.

**Methods:** We used our previously established syngeneic spinal metastases mouse model by injecting luciferin-transfected B16 melanoma cells into the common carotid artery. Following injection, mice were treated with everolimus, an inhibitor of the mammalian target of rapamycin (mTOR) complex, axitinib, a tyrosine kinase inhibitor, that blocks vascular endothelial growth factor receptors (VEGFR) 1-3, as well as placebo. Animals were followed-up daily by neurological assessment and by repeat *in vivo* bioluminescence imaging. With occurrence of neurological deficits, a spinal MRI was performed, and mice were sacrificed. The whole spine was dissected free and analyzed by immunohistochemical techniques.

**Results:** Overall survival was 23 days in the control group, significantly prolonged to 30 days (*p* = 0.04) in the everolimus group, and to 28 days (*p* = 0.04) in the axitinib group. While 78% of mice in the placebo group developed symptomatic metastatic epidural spinal cord compression, only 50% did so in the treatment groups. The mean time to manifestation of paralysis was 22 days in the control group, 26 days (*p* = 0.10) in the everolimus group, and 27 days (*p* = 0.06) in the axitinib group. Screening for spinal metastases by bioluminescence imaging on two different time points showed a decrease in metastatic tumor formation in the treatment groups compared to the controls. Immunohistochemical analysis confirmed the bioactivity of the two compounds: The Ki67 proliferation labeling index was reduced in the everolimus group and numbers of CD31 positive endothelial cells were reduced in the axitinib group.

**Conclusion:** Both, the mTOR inhibitor everolimus as well as antiangiogenetic effects by the VEGFR inhibitor axitinib showed potential to prevent and retard formation of symptomatic spinal metastases. However, the therapeutic efficacy was only mild in this experimental model.

## Introduction

Metastatic epidural spinal cord compression is a grave complication for cancer patients. If untreated, a significant spinal cord compression due to an epidural tumor mass, most frequently originating from the vertebra, will lead to relevant neurological deficits (1, 2). The metastatic process involves multiple cellular steps, such as intravasation, homing, extravasation, colony formation, and tumor growth (3). Spinal metastases occur in approximately 10 percent of cancer patients (4), they often represent the first manifestation of cancer (5), and the frequency of metastatic epidural spinal cord narrowing in cancer patients is estimated up to 8% depending on the primary tumor (6, 7). Treatment options are surgery and radiotherapy (8, 10). The gold standard in malignant extradural spinal cord compression is decompressive surgery within 24 h of diagnosis most often followed by radiotherapy (9, 10). While these treatment concepts are palliative in nature preventive therapies are not available and urgently needed.

In cancer treatment, targeted therapy has successfully developed in the last decade (11). However, an optimal pharmacological treatment specific for spinal metastatic tumors, especially in the recurrent stage or in a prophylactic setting, is not available yet. In our study, we chose two different targeted therapies, with the aim to show potential effects on spinal metastases formation and neurological outcome. Among others, inhibitors of the enzyme mammalian target of rapamycin (mTOR), as well as vascular endothelial growth factor receptor (VEGFR) inhibitors, are interesting targeting pharmaceuticals. The physiologic function of mTOR is regulating cell growth and metabolism, that is frequently highly activated in tumor cells (12), and the mTOR inhibitor and rapamycin derivate everolimus has shown promising anticancer activity in various tumor types (13, 14), with beneficial effects on bone turnover in bone metastases patients (15). Moreover, the mTOR signaling pathway is known to play a role in bone tissue signaling and in bone cancer (16). Another available targeted approach is inhibiting tumor angiogenesis, which is driven by vascular endothelial growth factor receptors (17). The tyrosine kinase inhibitor axitinib, currently approved for treatment of renal cell carcinoma (18), blocks VEGFR types 1, 2, and 3, leading to inhibition of tumor vascularization and growth (19).

In this study, we hypothesized to target the circulating tumor cell interactions with the bone vascular network by inhibiting the VEGFRs, and to block the mTOR signaling pathway to target the tumor colony formation and growth. We examined, whether targeting these signaling pathways is capable of preventing the formation of symptomatic spinal metastases in a murine hematogenous spinal metastases mouse model.

## Materials and Methods

### Substances

Everolimus/RAD001 as well as axitinib (both Biozol, Eching, Germany) were solved in an injection solution consisting of 30% polyethylene glycol (PEG), 65% H_2_O, 5% Tween 80, and 0.1% Dimethylsulfoxid (Sigma Aldrich, Schnelldorf, Germany). Everolimus was injected intraperitoneally (i.p.) with a concentration of 10 mg/kg body weight, and axitinib with a concentration of 25 mg/kg body weight. Placebo was the injection solution only. The i.p. injection volume was constantly 200 μl. Substances were stored at −20°C and freshly thawed immediately before use.

### Firefly Luciferase Transfection, Cell Culture and Spine Metastasis-derived Cell Line Establishment

B16-F1 murine melanoma cells (ATCC® CRL-6323) were infected with a FFLUC-eGFP-Puro vector construct (B16-luc) as described previously (20). Stable cell clone growth was maintained in culture flasks stored at 37°C, 5% CO_2_, as well as 95% humidity. Dulbecco's Modified Eagle Medium (DMEM) (Invitrogen, Carlsbad, USA) was supplemented with 10% heat-inactivated fetal calf serum (Thermo Fisher Scientific, Waltham, USA), 50 units/ml penicillin and 50 μg/ml streptomycin, as well as 5 μg/ml puromycin (Sigma Aldrich, Schnelldorf, Germany) for cell selection. The mB16-luc cell line was previously established by *in vivo* selection from an already grown B16-luc spinal tumor (20).

### Approval of Animal Experiments

All animal experiments were performed according to the UK Coordinating Committee on Cancer Research (UKCCR) Guidelines for the Welfare of Animals in Experimental Neoplasia (21, 22) and with the permission of the responsible local authorities from the Charité Universitätsmedizin Berlin and the LaGeSo Berlin.

### Retrograde Carotid Artery Injection

We used female, 20 weeks old, C57/B6J mice (Jax Stock No. 000664). As previously described (20), they were anesthetized (9 mg ketamine hydrochlorid/1 mg xylazine per 100 g body weight) intraperitoneally and the area of the operation was shaved and kept sterile during the operation. A longitude incision of the skin was made, and under the parotid gland, the common carotid artery was prepared and the vagus nerve was separated. The artery was ligated permanently distal and temporarily proximal to the aortic arch, in between incised, and a catheter (diameter 0.8 mm, filled with 0.9% sodium chloride solution) was inserted toward the aortic arch and fixed. The temporary ligature was opened, and a 100 μl cell suspension (1 × 10^5^ mB16-luc cells in serum-free DMEM) was slowly injected, followed by 100 μl 0.9% sodium chloride, for 1 min, respectively. Finally, the common carotid artery was completely disabled, the skin was stitched up, and mice woke up. They were randomly assigned to the placebo and the treatment groups. Despite fully common carotid artery occlusion, no neurological deficits, especially no pareses of limbs, were observed (20, 23).

### Treatment Schedule and Animal Examination

All mice gained access to water and a standard laboratory diet. Mice were treated with everolimus i.p. daily for consecutive 16 days in one group, with axitinib i.p. daily for consecutive 19 days in one group, as well as with placebo daily for consecutive 19 days in one group. Drug administration started on postoperative day 1, in order to ensure the identical tumor load in all groups at the start of therapy. Mice were examined daily, after occurrence of neurologic deficits like limb weakness, inability to run or paraplegia, or a bad health status, a spinal MRI was performed and mice were immediately sacrificed. The spine and brain were dissected, and frozen for further histological analysis.

### Bioluminescence Imaging

Imaging was performed on postoperative days 11 and 22 on every mouse with the IVIS Lumina II equipment (Caliper LS, Waltham, USA). During the procedure, mice were anesthetized with 2% isoflurane (Forene, Wiesbaden, Germany), and they were shaved along the spine for better imaging. D-luciferine (Caliper LS, Waltham, USA) was administered i.p. analogous to the manufacturer's protocol (30 mg/ml, 10 μl/g body weight) in order to activate cleavage by luciferase, selectively expressed by the tumor cells. After 5 min, mice were transferred into the imaging system. After an exposure time of 5 min, signals were measured as relative light units with Living Image 3.1 software (Perkin Elmer, Waltham, USA). A demarcated area above the spine was defined as one hot spot if it showed a clear signaling and could be distinguished from other neighboring signaling areas.

### Magnetic Resonance Imaging

MRI studies were performed at a seven Tesla small-animal system BioSpec 70/20 (Bruker BioSpin MRI GmbH, Ettlingen, Germany) with a BGA-12S HP gradient system and Bruker software Paravision 6.0.1. For imaging, a 1H−86 mm quadrature volume resonator and a receive—only 1H—phased array rat brain surface coil were used. During MRI examination, mice were placed on a heated circulating water blanket to ensure constant body temperature of 37°C. Anesthesia was maintained with 2.5–1.5% isoflurane delivered in an O_2_/N_2_O mixture (0.3/0.7 l/min) via a facemask under constant ventilation monitoring (Small Animal Monitoring & Gating System, SA Instruments, New York, USA). T2-weighted images of the whole mouse spine in the sagittal plane were made. For image acquiring, Paravision 6.0.1 software (Bruker, Billerica, USA) was used. For metastases number analysis, vertebral body as well as intraspinal tumors and tumors of the posterior column were counted.

### Spine Fixation and Preparation

For fixation, a whole animal perfusion was necessary. Mice were deeply anesthetized with 9 mg ketamine hydrochlorid/1 mg xylazine per 100 g body weight i.p. A thoracotomy was performed, a needle was placed into the left ventricle, and the right atrium was opened for outflow. Perfusion began with sodium chloride, until a bright liver indicated a good effect, and was completed by infusion with 4% paraformaldehyde (24). Whole spines were dissected, muscle tissue was completely removed, and samples were fixed in 4% paraformaldehyde solution on ice for 4 h. Then, spines were decalcified with 0.5M EDTA solution at 4°C for 24 h. After that, samples were stored in 20% sucrose and 2% polyvinylpyrrolidone for 24 h at 4°C. Finally, spines were embedded in a solution of 8 g gelatin, 2 g polyvinylpyrrolidone, and 20 g sucrose in 100 ml PBS, and stored at −80°C (25).

### Immunohistochemistry

Spine samples were transferred to a cryostat (Leica, Wetzlar, Germany), and cryosections were made at −20°C. Slides were dried and stored at −20 °C. For immunostaining, they were thawed and fixed in methanol (−20°C for 5 min) followed by 30 min incubation with blocking buffer (1% casein in PBS). The primary antibodies against Ki67 (Thermo Fisher Scientific, Waltham, USA) and CD31/PECAM-1 (BD Pharmingen, Franklin Lakes, USA) as well as FITC-conjugated and Cy3-conjugated secondary antibodies (Dianova, Hamburg, Germany) were added for 2 h, respectively. Cell nuclei staining was performed by incubation with 4',6-diamidino-2-phenylindole (1:100 in PBS, Roth, Karlsruhe, Germany) for 5 min. Slides were covered with Immu-Mount (Thermo Fisher Scientific, Waltham, USA). By fluorescence microscopy, 30 high-power fields from three different spinal metastases per group were randomly chosen, Ki67 positive and negative stained cell nuclei were compared by percentage with ImageJ software (NIH, Bethesda, USA), CD31 positive endothelial cells were counted and subsequently analyzed. Negative controls without processing primary antibodies did not display any specific immunoreactivity.

### Statistical Analysis

Data were shown as mean ± standard deviation. The Mantel-Cox test (logrank test) was used for survival analysis as well as analysis for neurological deficit occurrence. For immunohistochemical analysis, the Kruskal–Wallis test was used to test for significant differences among groups (*p* = 0.009 for the Ki67 analysis, *p* < 0.001 for the CD31 analysis), and the Mann–Whitney *U*-test was applied for group comparisons. Differences with *p* < 0.05 were considered as statistically significant. Statistical differences were calculated with SPSS software (IBM, Armonk, USA) as well as with GraphPadPrism software (La Jolla, USA).

## Results

### Spinal Epidural Metastases Development

**Figure 1:**
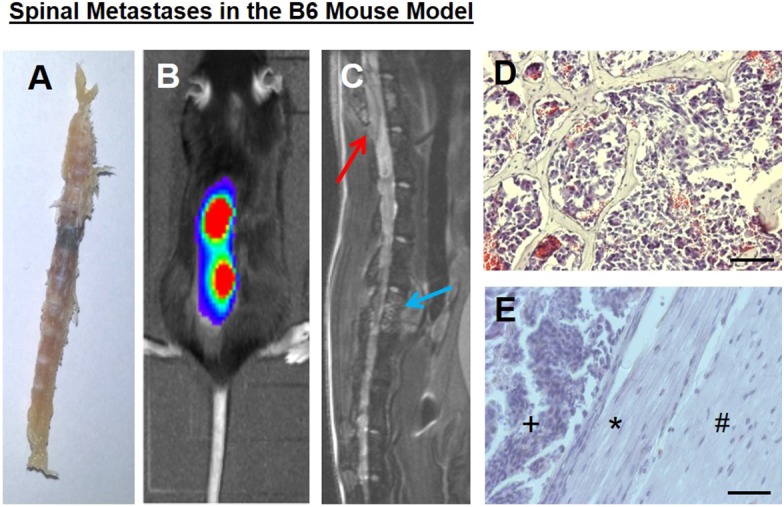
**(A)** A spine column explant with a black melanoma metastasis. **(B)** Correlating to bioluminescence signals, a **(C)** spinal sagittal plane MRI shows a vertebral body metastasis, blue arrow, as well as a metastasis with epidural spinal cord compression, red arrow. **(D)** Tumor cells infiltrated the trabecular bony structure (representative hematoxylin and eosin staining). **(E)** Intraspinal epidural tumor (+), dura impression (*), nervous tissue (#). Bars indicate 100 μm.

In our mouse model, bony metastases in the spine with epidural spinal cord compression developed within 2–3 weeks, as previously shown (20, 26), without solid brain metastases formation, at least in the corresponding time periods, which could also lead to neurological deficits. Spine column explants already macroscopically showed black melanoma metastases (Figure 1A). Both bioluminescence imaging (Figure 1B) and spinal MRI (Figure 1C) allowed to assess spinal metastatic burden *in vivo*. Conventional histology illustrated epidural metastatic spinal cord compression and demonstrated tumors that infiltrated the trabecular vertebral bone structure and compressed the dura and spinal cord (Figures 1D,E).

### Prolonged Overall Survival

**Figure 2:**
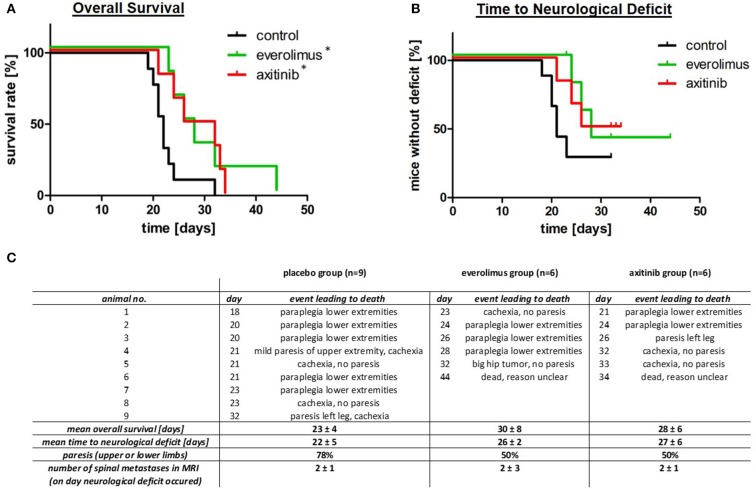
Overall survival, time to neurological deficits as well as clinical symptoms. **(A)** Kaplan-Meier survival plots, comparing the control group (black), everolimus group (green), and axitinib group (red) with a significantly (*p = 0.04) lower mortality in the treatment groups. **(B)** Time to neurological deficit, defined by extremity paresis, with a tendency to a prolonged time to this event during everolimus or axitinib treatment, but without reaching statistical significance (everolimus group *p* = 0.10, axitinib group *p* = 0.06). **(C)** This table summarizes data of all experimental groups. The day number describes the postoperative day, when the respective event leading to death occurred.

Treatment with everolimus or axitinib prolonged overall survival of tumor bearing mice by up to 30% when compared to placebo (Figure 2A). In the control group, mean survival was 23 ± 4 days compared to 30 ± 8 days in the everolimus group and 28 ± 6 days in the axitinib group (Figure 2C). The differences compared to the placebo group were statistically significant (*p* = 0.04).

### Effect on Neurological Deficit

In contrast to survival, the effect on formation of spine metastasis was less pronounced. Seventy-eight percent of mice in the control group showed paralysis of upper or lower limbs, compared to 50% in the treatment groups (Figures 2B,C). MRI confirmed that all of these episodes of neurological deterioration were related to a spinal metastasis. The time to development of these neurological deficits was, however, not statistically different. Mean time to neurological deficit was 22 ± 5 days in the control group. In the everolimus group, time to paresis was 26 ± 2 days (*p* = 0.10) and in the axitinib group, time to neurological deficit was 27 ± 6 days (*p* = 0.06; Figure 2C).

### Spinal MRI Directly After Onset of Neurological Deficits

After onset of a neurological deficit, a whole spinal MRI was performed and thereafter, mice were sacrificed. The numbers of spinal metastases were counted on the spinal MRI in sagittal cuts. We found a mean metastases number of 2 ± 1 in the control group, 2 ± 3 in the everolimus group, and 2± 1 in the axitinib group, respectively (*p* = 0.91; Figure 2C).

### Bioluminescence Tumor Visualization

**Figure 3:**
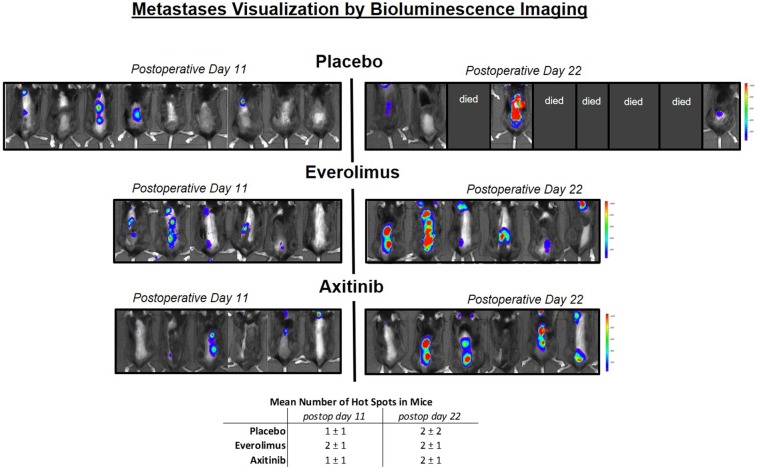
Metastases visualization by *in vivo* luminescence imaging. Left side shows mice on postoperative day 11, right side day 22. Blue color implies low signal intensity, red color high signal intensity, as relative light units in the color scale. In the placebo group, 5 mice already died before measurement on postoperative day 22 could be performed, in the treatment groups, all mice could be measured. A heightened signal intensity over time of metastases correlating with tumor growth could be shown in all mice.

We conducted a bioluminescence spine metastatic tumor screening after postoperative days 11 and 22 (Figure 3). Areas of spinal hot spots correlated well with the results of MR imaging (Figures 1B,C). After 11 days, we observed similar numbers of metastases per mouse in the experimental group (control, everolimus and axitinib group 1 ± 1, 2 ± 1, 1 ± 1, respectively; *p* > 0.05), as well as similar numbers on postoperative day 22 (2 ± 2, 2 ± 1, 2 ± 1, respectively; *p* > 0.05). A heightened signal intensity over time of already existing metastases, indicating tumor growth, could be measured in all mice.

### Histological Analysis

**Figure 4:**
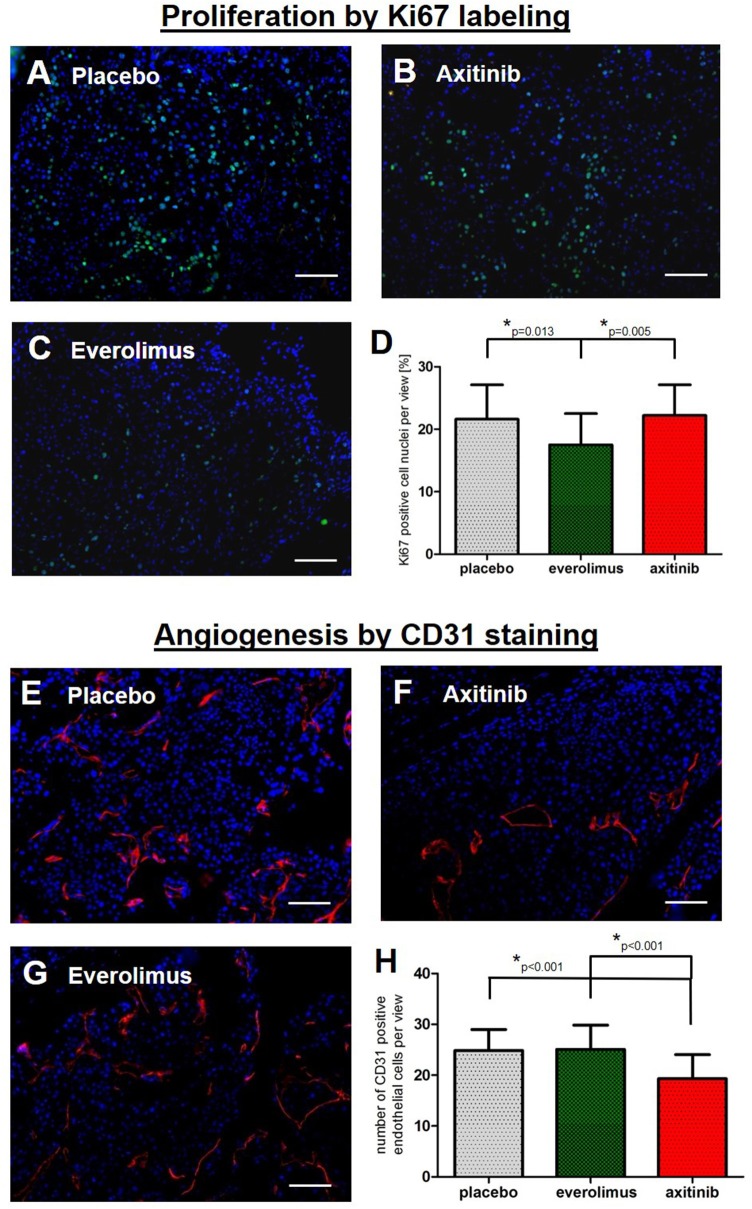
Immunohistochemical analysis. Graphs show the summary of data from all randomly chosen high-power fields from 3 different spinal metastases per group. **(A–D)** Fluorescence-provided Ki67 stainings of tumor cell nuclei in the placebo group, axitinib group and everolimus group. The fraction of proliferating cells was lowered in the everolimus group (*p* = 0.013). **(E–H)** Numbers of endothelial cells marked by CD31 immunostainings revealing a lowered number of endothelial cells as well as larger tumor vessels in axitinib treated mice (*p* < 0.001).

Next we aimed at understanding the mild effects in the treatment arms. To reveal a possible treatment effect on metastases proliferation, we examined spinal tumor tissue by Ki67 staining. In the control group, there was a mean of 22 ± 6% of stained cell nuclei, compared to 18 ± 5% in the everolimus group (*p* = 0.013), and 22 ± 5% in the axitinib group (Figures 4A–D). To assess anti-angiogenic activities, we stained for the endothelial cell marker CD31. There was a mean number of endothelial cells per high-power field of 25 ± 4 cells in the controls, of 25 ± 5 cells in the everolimus group, and of 19 ± 5 cells in the axitinib group. The difference between the axitinib group and the control group was statistically significant (*p* < 0.001). Furthermore, we observed larger vessels within spinal metastases of axitinib treated mice (Figures 4E–H).

## Discussion

In the present study we have shown that in our mouse model bony metastases developed within the spine causing epidural spinal cord compression, and it is possible to monitor the occurrence and numbers of spinal metastases by bioluminescence imaging or MRI. The principal novel findings are a significant prolonged overall survival of mice treated with the mTor inhibitor everolimus or after therapy with the VEGFR blocker axitinib, as well as a prolonged time to neurological deficit, triggered by a spinal metastatic tumor, in both treatment modalities.

Malignant spinal cord compression caused by spinal metastases can lead to neurological symptoms up to paraplegia with a serious reduction in quality of life. Generally, tumor metastases are located most frequently in the lungs and liver, but then followed by the skeletal system including the spine (27). Malignant spinal cord compression can indicate an advanced tumor stage, verified by the observation of a limited overall survival after diagnosis (2, 28).

In the acute situation of a neurological deficit, surgery is the therapy of choice, in order to improve or retain neurologic function. If a patient is not operable, radiotherapy combined with corticosteroids can be initiated (9). Most often, a resection is accompanied by local radiotherapy and, depending on the tumor stage, systemic chemotherapy (2). Nevertheless, the local recurrence rate after treated symptomatic spinal metastases is about 7 to 14 percent (2, 29, 30). To focus on these patients, targeted therapies as an adjunct to the standard treatment seem to be promising, with the aim to prevent as well as treat these tumors, and to stop the growth of other possibly existing metastatic tumors within the spine.

Therefore, we previously established a hematogenous spinal metastases mouse model to examine basic principles of spinal metastases formation (20). We adapted a bone metastases mouse model (31) by injecting tumor cells into the common carotid artery to avoid the pulmonary filter system, so that tumor cells spread into the arterial blood leading to an endogenously spinal metastases formation process. It is assumed that tumor cells are reaching the vertebral bodies by a hematogenous route (32), and arterial embolization seems to be the most relevant mechanism of metastasis development (33). The distribution of spinal metastases is about 60% in the thoracic, 25% in the lumbar, and 15% in the cervical spine (1, 6, 34), with similar distributions in our spinal model shown by bioluminescence monitoring, indicating a matchable system regarding to the mechanism of spinal metastases formation, which is, however, still not fully understood.

In our model, we used murine melanoma cells (35), obtaining the advantage to use immunocompetent mice, and to retain potential influences of the immune system. For cell injections, we used our stable cell line mB16, established from solid grown B16 spinal metastases in this model by *in vivo* selection, with a significantly higher potential to metastasize to the spine (20). The advantage is that mice, after tumor cell injections, generally did not suffer from significant tumor burden in other organs like the lung, liver or brain, and a nearly spine-specific tumor model was available.

One aim of our study was to confirm this preclinical model as valid for therapeutic studies regarding spinal metastases formation. Moreover, we applied two different targeted therapy approaches with the following hypothetical impacts: On the one hand, we wanted to target the potential circulating melanoma cell interactions with the bone vascular network by inhibiting the VEGFRs, in order to target the vascular formation step in the metastases origination cascade. On the other hand, we blocked the mTOR signaling pathway to potentially target tumor bone colony formations and spine metastasis growth, as the last step in metastasis bone formation.

We could find a significantly prolonged overall survival in both therapy groups. Placebo treated mice lived 23 days on average, after everolimus or axitinib therapy, mice survived 7 (prolonged by 30%) and 5 days (prolonged by 25%) longer, respectively. This indicates a general antitumor effect in the systemic compartment. In order to assess symptomatic spinal metastases, we focused on acute neurological deficits, e.g., limb paresis. A causal spinal cord compression was verified by MRI. Here, we found a 18% prolonged time after mTOR inhibition by everolimus, and a 23% prolongation after VEGFR blockade via axitinib therapy, until neurological deficits due to spinal cord compression occurred. We state that our model serves as a valid system for examining therapy responses on spinal metastases formation, and for evaluation of the neurological outcome.

The luminescence reporter imaging for tumor visualization served to detect possible dynamic effects on spinal metastases formation and growth *in vivo* (36). Between two different time points during therapy, on postoperative days 11 and 22, we did not detect any significant change in the numbers of spinal tumor hot-spots among groups. In the spinal MRI, we detected similar numbers of metastases in comparison with the bioluminescence method on postoperative day 22, considering the different time points the methods were applied. The bioluminescence screening was used on two defined time points, whereas the spinal MRI was performed directly after the occurrence of a neurological deficit in order to show the responsible spinal tumor, which apparently began on different dates for each mouse. Though, we presume a higher increase in numbers of novel developing spinal tumor hot-spots during axitinib therapy, in contrast to a continuous growth of already early existing metastases in the everolimus group. More frequent time intervals could reveal clearer effects, and being aware of the limited validity, i.a. the small differences and the small number of mice in the placebo group on the second time point, it seems possible that VEGFR inhibition could delay the time point of the development of solid spinal metastases, similar to a prophylaxis, maybe due to angiogenesis inhibition after tumor cells are entrapped passively (26). For example, in prostate tumors, the expression levels of VEGF and VEGFRs were higher at the bone metastases site compared to the primary tumors, indicating the importance of angiogenesis in metastasis development to the bone (37), and vascular factors could encourage the nesting of tumor cells in the bone (38). Angiogenesis inhibition could be revoked by tumor cell adaptation processes, and the antiangiogenic effects could be pronounced in early stages of metastases formation. At this time point, tumors could be most accessible to targeted VEGFR inhibition, with the focus on vessel formation, delaying the process of building their own blood supply. This theory could be addressed in future projects.

Further immunohistochemical analysis showed that in late stage spinal metastases, the proliferation of tumor cells was slightly lowered after mTOR inhibition, detected by a decrease in the Ki67 labeling index. VEGFR inhibition did not influence the tumor proliferation rate, but reduced the number of endothelial cells, and larger vessels were observed, indicating a normalization of the tumor vasculature, and possible antiangiogenic effects. This indicated two different impacts on malignant tumor properties after two different targeted treatments against spinal metastases, also confirming the bioactivity of the two applied compounds. Here, the mild histological effects could not finally confirm the expected mode of actions as a therapy target, and our applied therapy did not elicit a significant impact on the spinal tumor compartment, though proved significant prolongation of the overall survival as well as prolongation of the occurrence of neurological deficits.

In this study, we demonstrated a feasible preclinical mouse model suitable for investigating targeted therapy approaches against metastatic spinal cord compression. It is important to better understand the molecular mechanisms in spinal metastases formation and further experimental data is necessary, with the general aim to offer patients individualized therapy options.

## Data Availability Statement

The datasets generated for this study are available on request to the corresponding author.

## Ethics Statement

The animal study was reviewed and approved by Landesamt für Gesundheit und Soziales (LAGeSo) Berlin, Turmstraße 21, 10559 Berlin, Germany.

## Author Contributions

PV and TK designed the study. TK carried out and analyzed the experiments and wrote the manuscript. AP, TB, and MC gave support to experiments. PV and TK revised the manuscript. All authors approved the final version.

### Conflict of Interest

The authors declare that the research was conducted in the absence of any commercial or financial relationships that could be construed as a potential conflict of interest.
